# RECIST 1.1 and lesion selection: How to deal with ambiguity at baseline?

**DOI:** 10.1186/s13244-021-00976-w

**Published:** 2021-03-18

**Authors:** Antoine Iannessi, Hubert Beaumont, Yan Liu, Anne-Sophie Bertrand

**Affiliations:** 1 Median Technologies, 06560 Valbonne, France; 2 Centre Hospitalier Princesse Grâce, Monaco, 98000 Monaco

**Keywords:** RECIST, Clinical trials, Oncology, Therapeutic response

## Abstract

Response Evaluation Criteria In Solid Tumors (RECIST) is still the predominant criteria base for assessing tumor burden in oncology clinical trials. Despite several improvements that followed its first publication, RECIST continues to allow readers a lot of freedom in their evaluations. Notably in the selection of tumors at baseline. This subjectivity is the source of many suboptimal evaluations. When starting a baseline analysis, radiologists cannot always identify tumor malignancy with any certainty. Also, with RECIST, some findings can be deemed equivocal by radiologists with no confirmatory ground truth to rely on. In the specific case of Blinded Independent Central Review clinical trials with double reads using RECIST, the selection of equivocal tumors can have two major consequences: inter-reader variability and modified sensitivity of the therapeutic response. Apart from the main causes leading to the selection of an equivocal lesion, due to the uncertainty of the radiological characteristics or due to the censoring of on-site evaluations, several other situations can be described more precisely. These latter involve cases where an equivocal is selected as target or non-target lesions, the management of equivocal lymph nodes and the case of few target lesions. In all cases, awareness of the impact of selecting a non-malignant lesion will lead radiologists to make selections in the most rational way. Also, in clinical trials where the primary endpoint differs between phase 2 (response-related) and phase 3 (progression-related) trials, our impact analysis will help them to devise strategies for the management of equivocal lesions.

## Key points

- When using RECIST, baseline tumor selection is all-important.
- The selection of equivocal lesions at baseline is detrimental to patient assessment.
- A strategy can be designed to limit the impact of including equivocal lesions at baseline.

## Background

Baseline oncologic evaluations are critical procedures as target selection determines the quality of the overall review. The RECIST workgroup published a method and recommendations for the selection of targets and non-targets in order to obtain accurate, reproducible and representative information regarding disease extension [1]. However, this selection and categorization process varies from one reader to another, one reason for this variability being the equivocal status of some lesions.

In oncologic follow-up, equivocal lesions can be defined as lesions for which the radiologist is unsure whether they effectively correspond to the designated malignant disease.

Indeed, a given radiologic semiology is not specific to a single malignant etiology and some abnormalities may be artifactual or linked to transient nonmalignant disease (e.g., adverse effect, inflammation). In addition, ground truth (i.e., biopsy) is often unavailable for such images.

In practice, radiologists might, or might not, include equivocal lesions in their initial RECIST pool of lesions. In this study, we define these two approaches used by radiologists as non-conservative and conservative, respectively. This mode of decision-making is therefore subjective.

The goal of this paper is to provide insights for radiologists faced with equivocal baseline abnormalities and to raise awareness of the potential risks arising from such situations regarding the outcome of clinical trials using the RECIST evaluation technique.

In short, we aim to answer the following question: “When radiologists are confronted with a tumor which they cannot identify with certainty as a malignancy, should they register it in any case in order to avoid omitting a lesion when evaluating the initial disease?”.

In the specific context of clinical research, we will first recapitulate the problem raised by the variability of baseline assessments. Second, we will document the notion of equivocal lesions and discuss the factors contributing to their ambiguity. Third, we will analyze the risks resulting from the inclusion of non-malignant lesions in the pool of targets relative to RECIST longitudinal assessment. Lastly, we will suggest recommendations for baseline target selection in the context of clinical trials.

## Recap on baseline variability as a risk factor in clinical trials

### Baseline selection variability

The RECIST guidelines allow some measure of freedom in the selection of targets.

**Fig. 1:**
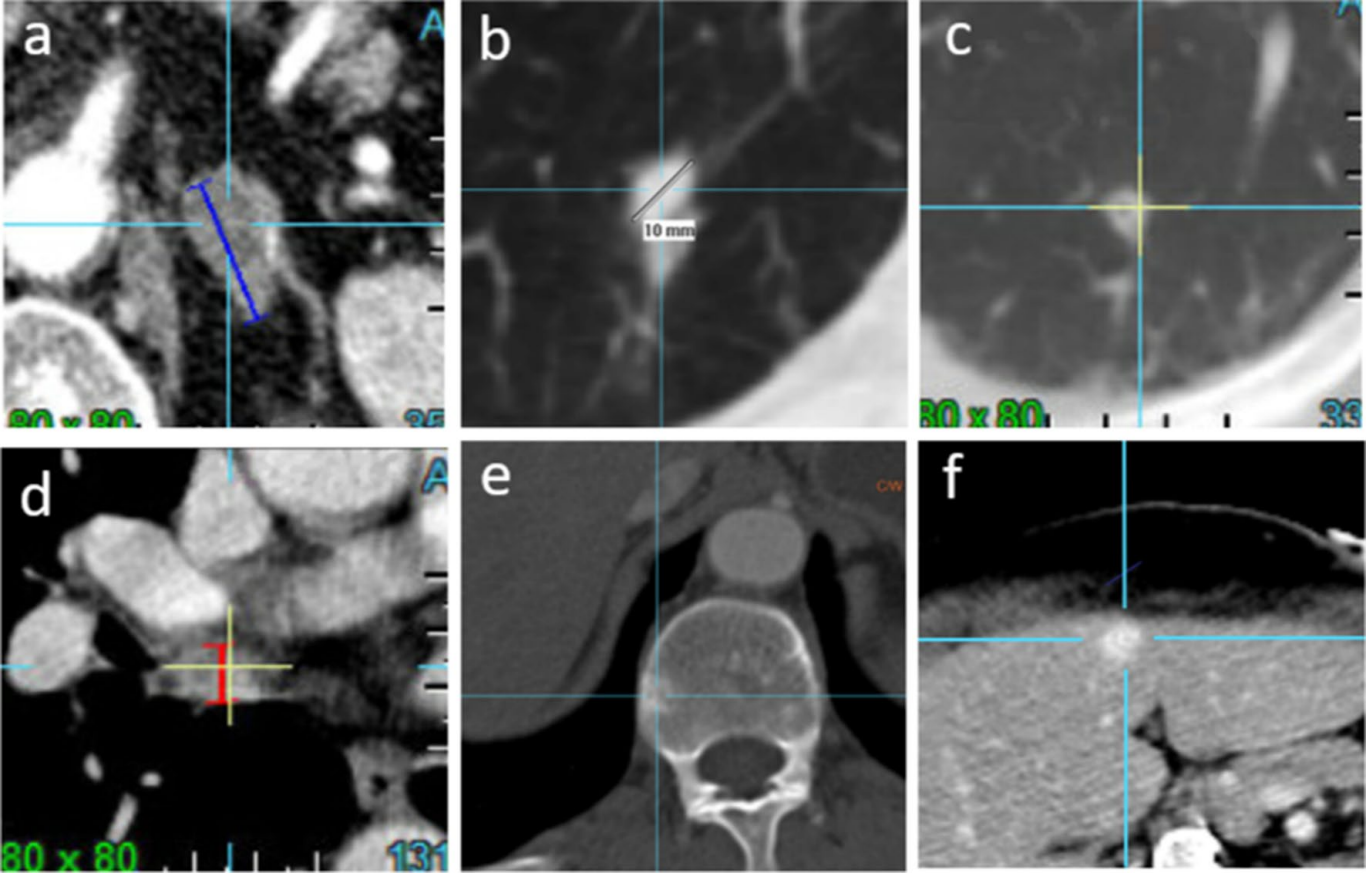
Views of ambiguous lesions selected at baseline evaluation (derived from RECIST blinded double-reading central review database). **a** measurable adrenal nodule believed to be a metastatic lesion from the primary lung cancer (follow-up: revealed to be a benign incidentaloma); **b** 10 mm nodule with ground glass in a context of metastatic colon (follow-up: revealed to be malignant and responded like the rest of the TLs); **c** non-measurable well-defined lung micro-nodule in a context of head-neck cancer follow-up (follow-up: revealed to be stable and most probably benign); **d** non-measurable supra-centimetric mediastinal lymph node in a context of colon cancer (follow-up: revealed to be stable supra-centimetric); **e** low conspicuity of a blastic bone lesion (follow-up: revealed to be a metastatic lesion confirmed by sclerotic healing changes); **f** hypervascular centimetric nodule in the liver in the context of metastatic colon cancer (follow-up: revealed to be stable and probably unrelated to the cancer)

**Fig. 2:**
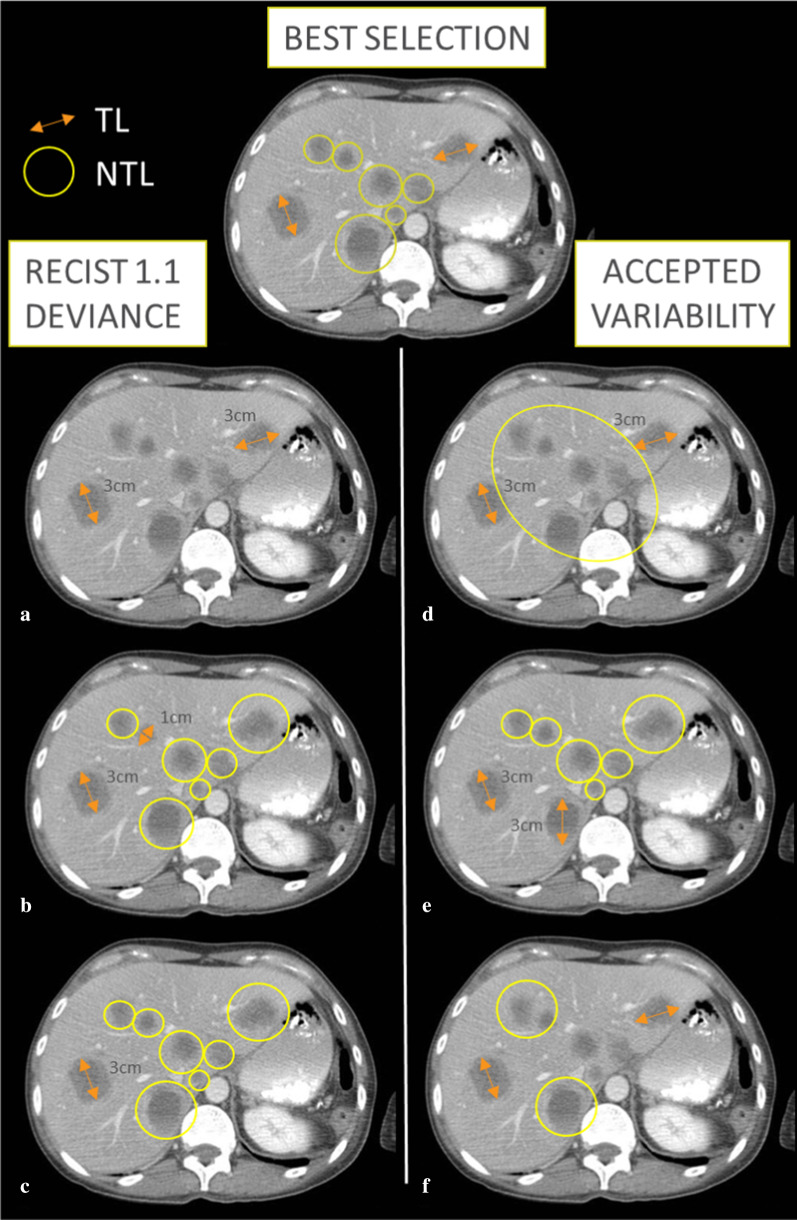
RECIST 1.1 baseline selection accepted variability and errors. TL: Target Lesions are measured by double arrows, NTL: Non-Target Lesions are circled. This patient had a disease limited to the liver. We illustrate 3 different types of baseline lesion selection that can be considered as errors and that deviate from RECIST guidance: **a** if no NTL have been selected; **b** if the smallest Target Lesions have been selected instead of the largest; **c** if only 1 TL has been selected while 2 were measurable. We illustrate 3 different lesion poolings that can be considered as variations from the ideal selection without deviating from the RECIST guidelines: **d** if NTL lesions are grouped inside an organ; **e** if different TL are chosen within the largest lesions; **f** if fewer NTL are selected considering the evaluation as categorial and qualitative even though, preferably, they should all be recorded but practically it is not always possible.

In addition to equivocal lesions as shown in Fig. 1 for a given disease presentation, there exists a wide variety of lesion selection patterns, all complying with RECIST recommendations (Fig. 2) [1, 1].

RECIST advocates choosing lesions at all disease sites and a maximum of 2 target lesions (TL) per organ. Although conforming with the guidelines, some radiologists would probably consider it more relevant to select a larger number of TL, while others would select fewer TLs and advance sound reasons for their choice [3].

In addition, the measurability criteria stipulated by RECIST for selecting TL include not only size (≥ 10 mm for non-nodal lesions) but also measurement reliability and reproducibility. These last two items are subjective and leave room for individual readers to select their own targets. Moreover, reproducibility is an unforeseeable feature of TLs since it can only be confirmed once the longitudinal analysis is complete.

The factors responsible for the variability of baseline selection patterns are listed in Table 1 [4–6].

**Table 1:** Factors of inter-reader variability for selecting lesion at baseline following RECIST 1.1 guidance

Category of targets	TL	NTL
Factors of inter-reader variability	Size measurement variability for small centimetric lesions (10 mm measurability threshold) Confidence of measuring (e.g., Ill-defined lesions, artefacts) Reproducibility of measurement is not ensured (e.g., position of the lesion, digestive tract lesion) Equivocal malignancy (i.e., benign lesion mimicking malignant lesion, small undetermined lesions)	Several grouped lesions recorded as one NTL Undefined number of NTL Equivocal malignancy (i.e., benign lesion mimicking malignant lesion, small undetermined lesions)

### RECIST endpoints aligned with clinical trials outcomes

The RECIST endpoints are used in drug development as surrogate imaging biomarkers.

In clinical trials, and depending on the phase of development, radiographic assessment is performed to generate an endpoint related to progression of the disease and/or response to therapy**.** Moreover, this surrogate endpoint is critical to the study outcome if it is taken as the primary endpoint. To ensure correct assessments, the FDA recommends procedural and methodological standards, including imaging-related recommendations [7]. The main objective of these recommendations is to reduce evaluation bias and guarantee comparable evaluation standards.

A blinded Independent Centralized Review (BICR) is the recommended option. The procedure for reading might involve two radiologists. For these double reviews, an adjudicator is needed if the readers generate discordant assessments. The discrepancy rate is a key indicator for monitoring ongoing clinical trials using radiology [8, 8].

Baseline target selection is a critical process in quality assessment as differences in baseline target selection are known to be major causes of discrepancy [10].

For this reason, the absence of a strategy designed to reduce equivocal situations at baseline or the lack of clear reading rules when encountering such situations entails two risks for clinical trials.

- First, during a double reading paradigm, the selection of equivocal lesions at baseline contributes to inter-reader variability and thus increases the discrepancy rate. Subsequently, it increases the cost of the trial due to more frequent recourse to the adjudicator.
- Second, if the equivocal lesion turns out to be non-malignant, it will directly impact the study endpoint by reducing its sensitivity to the drug response [11].

In the sections below, we will describe measures aimed to prevent and cope with equivocal lesions during RECIST baseline evaluation.

## What makes lesions equivocal at baseline?

Some radiological abnormalities might not be easy to characterize for different reasons detailed in Table 2. We have identified 2 families of causal factors, first on the radiological features and second on the clinical information.

**Table 2:** Causal factors of equivocal lesions during the oncologic assessment

Related type of cause	Risk factors for ambiguous lesion
Technical	Poor quality of the examination Artefact: kinetic or any other
Protocol	Absence of imaging without contrast-agent Absence of triphasic acquisition for liver analysis
Contrast	Contraindication of contrast-agent
Lesion	Small size (non-specific very small lesions) Low conspicuity (small size, ground glass…) Radiological semiology in the malignancy/benign overlap zone
Study design (blinded, centralized review)	Lack or imprecise data on previous local therapy or biopsy

### Uncertainty due to the radiological features

The involutivity criterion is a very discriminating source of information to confirm whether an equivocal lesion is malignant or not [2]. However, at baseline, the radiologist might have no access to previous examinations to compare with. Such situations can occur, if the patient has not undergone a previous examination or if the results were not forwarded to the radiologist.

In such cases, it is not possible to determine whether an equivocal lesion was pre-existing and should be considered as a scar or a benign lesion, or whether it was not visible and should be considered as a malignant lesion or an artefact.

### Uncertainty caused by censored clinical data

A BICR might increase the rate of equivocal situations by limiting the information available to the radiologist compared with on site or unblinded evaluations. In general, inadequate information may prevent clarification of an equivocal lesion.

For example, biopsy-derived information is easily retrievable on site. In contrast, during a central review, the information is not always communicated to the reader. On site, this information would enable the practitioner to select a nodule if the biopsy is found to be positive whereas, during a central review, if the reader is blinded to the result the nodule will remain equivocal.

Another common example is a previously non-communicated radiated zone or one not fully explained to the radiologist and which thus can lead to the possible inclusion of an inactive scar in the pool of chosen targets.

## To target or not to target, that is the question…

Given that the status of some equivocal lesions at baseline cannot be determined, our aim was to analyze the risk of strategizing a non-conservative versus a conservative approach to target selection.

The intuitive argument for choosing a non-conservative approach is that recording as many visible lesions as possible at baseline will increase the accuracy of the measurements of disease evolution. Additionally, one could assume that the intrinsic variability of the RECIST guidelines mitigates the risk of misclassification, even if some non-malignant lesions are selected at baseline.

Conversely, a cautious conservative attitude would avoid selecting questionable malignant lesions and would lead to a failure to identify certain malignant lesions at follow-up.

The impacts of each strategy are summarized in Table 3.

**Table 3:** Impacts of non-malignant lesion selection within TL or NTL group according to the primary endpoint captured by the assessment

Baseline equivocal lesions pooled as	Remaining lesion	Response-Related Endpoint impact	Progression-Related Endpoint impact
TL	At least 1 other TL With or without NTL	Prevents a CR Limited impact on PR only if %NMTL < 20%	Delayed (or no) DOP Limited impact only if %NMTL < 20% and no initial response
	No other TL No other NTL	Simulates a SD and prevents a CR*	No impact
	No other TL At least 1 NTL	Prevents a CR	No impact
NTL	With or without TL	Prevents a CR	No impact

*The second reader would assess a baseline status of Non-Disease raising an eligibility issue if the measurability of the disease is an inclusion criterion

### Equivocal lesions recorded as target lesions

The risk in including a non-malignant Target Lesion (TL) at baseline is that some lesions will remain stable and will not contribute to modifying (increase or decrease) the total burden.

Indeed, we hypothesize that all lesions related to the same disease will shrink or progress relatively homogenously. Both paradoxical response and unstable equivocal lesions will be discussed below.

Based on these assumptions, the impact of equivocal lesions within the baseline target selection will depend on the percentage of Non-Malignant Target Lesions (NMTL) compared to the rest of the lesions contributing to the Sum of Diameters (SoD).

Indeed, this selection bias will result in changes in the threshold of response and progression for the initial unequivocal targeted lesions.

#### Quantification of the impact

A mathematical simulation was performed to quantify the impact of an inappropriate selection of NMTL within the selected target pool at baseline. We hypothesized that at least one unequivocal target lesion was also selected.

Our simulation relied on the Eq. 1 below (details are provided in the annex).

1 $$\beta = \frac{\Delta }{{100 \times \left( {1 - \alpha } \right)}} + 1$$

where $\Delta$ is the percentage of change between baseline and a given follow up time-point, $\alpha$ is the proportion of NMTL in the total SoD at baseline, $\beta$ is the required percentage change of malignant TL proportion to reach $\Delta$ at the given follow up time-point.

**Fig. 3:**
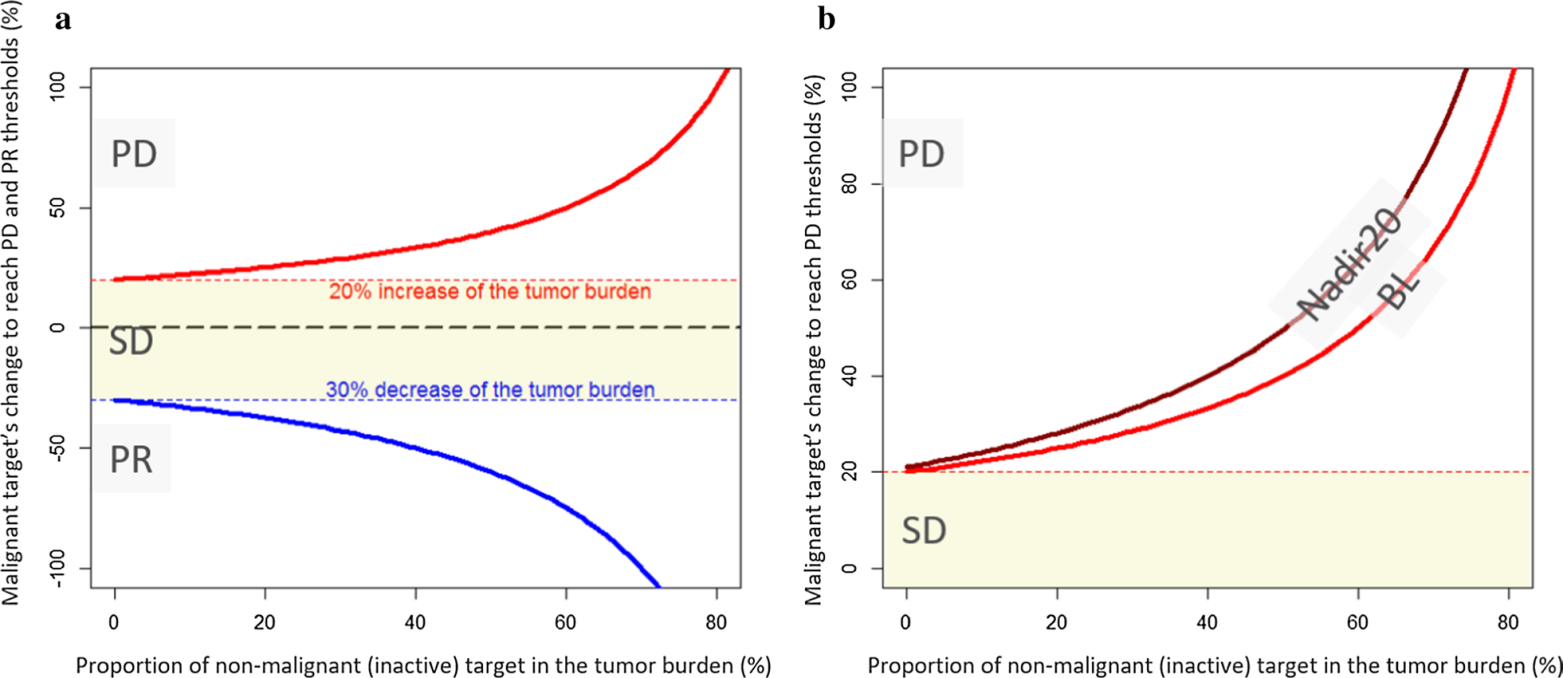
Mathematical simulation of the proportional change of real malignant target lesions (TL) to trigger progression or response according to RECIST 1.1 thresholds in accordance with the proportion of non-malignant inactive lesions within the tumor burden. **a** For the Sum of Diameter thresholds corresponding to a progressive disease + 20% (red) and to a partial response − 30% (blue), we plotted the proportional change of the malignant part of the tumor burden at a given time point with respect to the proportion of inactive tumors in the tumor burden at baseline. We noted that the curve steepens from a proportion of about 50% of inactive lesions while the impact is limited to 20% of NMTL pooled in the tumor burden. **b** For the Sum of Diameter thresholds corresponding to a progressive disease, we simulated the needed change of malignant target to trigger a progression from baseline (red) (similar to **a**) and nadir20 (dark red) after a 20% PR. This scenario decreases even more the sensitivity of the RECIST assessment to capture progression

Figure 3a shows that RECIST guidelines mitigate the risk of selecting NMTL when they do not constitute more than 20% of the total SoD. If the targets are approximately the same size, then including one equivocal lesion out of the 5 targets, as recommended by RECIST 1.1, will not have a major impact. Indeed, the remaining target threshold for Progressive Disease (PD) or Partial Response (PR) is not extremely different, i.e. respectively + 25% (vs 20%) and − 37% (vs-30%). However, as the NMTL forms part of the target pool, a complete response will be prevented from occurring.

In addition, we simulated a frequent type of response pattern when a response is observed followed by progression. Figure 3b shows the relative percentage of NMTL after a partial response. As can be seen, this type of response pattern increases the induced NMTL selection bias within the baseline TL pool. Using the example quoted above, even if only 20% of the burden corresponded to NMTL at baseline, this percentage increases to + 28% after a first PR.

These mathematical simulations serve as real incentives to avoid risk-taking when selecting an equivocal lesion as a target lesion, especially when it is larger than the unequivocal targets or if there are few targets in all. Moreover, the sensitivity for capturing progression is even more impacted after a first response.

#### Specific case of single target lesions

In this case, we cannot use the mathematical simulation to quantify the impact. However, in the overall assessment, the situation differs according to the primary endpoint evaluated.

- Concerning the progression-related endpoint, if the single target is non-malignant, no impact should occur. The size of this NMTL should remain stable during the longitudinal analysis. The progression status is triggered either by the non-target lesion category progression or the appearance of a new lesion. In this case, having a conservative or non-conservative strategy at baseline does not impact the date of progression.
- In contrast, concerning the response-related endpoint, a NMTL lesion will inevitably prevent a complete response, as above.

### Equivocal lesions recorded as non-target lesions

Instead of recording an equivocal lesion as a TL, one strategy could be to gather all equivocal lesions within the non-target lesions (NTL) category. This would not bias the objective quantification but still maximize the chance of capturing disease evolution by recording all suspect lesions.

Indeed, the overall assessment of NTL for PR allows more flexibility as RECIST states that an unequivocal increase of these lesions should always be taken into account in conjunction with the TL response.

- For this reason, a slight increase cannot trigger an overall PD. If the primary endpoint of the evaluation is detecting progression, the non-conservative strategy will minimize the risk of missing the PD.
- However, when the primary endpoint of the study is the Best Overall Response, promoting the non-conservative strategy might prevent an overall CR status and increase the discrepancy based on PR/CR assessment between readers in a study with a double-reading paradigm.

## Risk and mitigations

RECIST guidelines provide detailed recommendations on managing an equivocal progression based on ambiguous new lesions or NTL. In a previous edition of RECIST, it is suggested that this situation should be clarified using another later assessment [11]. Unfortunately, management of equivocal baseline lesions is not described in the initial RECIST guidelines [1] whereas the baseline selection of TL and NTL appears to be essential.

### Preventive strategies

As previously mentioned, equivocal lesions may be revealed at the initial baseline RECIST evaluation and these lesions have diverse causal factors (Table 2). Actionable strategies to prevent their occurrence are linked to image acquisition and to study design:

- Before the images are submitted to the radiologist, the technicians play an important role in achieving quality control by ensuring the absence of artefacts and compliance with the acquisition protocol.
- The acquisition protocol, comprising both non-contrast and multiphasic acquisition at baseline, should be the rule in order to facilitate differentiation between liquid/solid and malignant/benign enhancement profiles (e.g. liver angioma). This recommendation applies to all types of cancer especially when the trial does not include access to previous exams. According to the protocol, scintigraphy or multiparametric MR modality could be performed for baseline examination to improve characterization of possible bone, brain, liver anomalies. Off-protocol images performed on-site, e.g. additional MR examination to clear up small equivocal lesions, should also be collected and provided to the central review.
- When the RECIST evaluation is blinded or performed during a centralized review, the ability to assess a previous scan before baseline would help clarify certain potentially equivocal lesions. Previous radiation therapy information and the results of any biopsy should also be provided accurately to the radiologist to help him/her select the target more confidently at baseline. Some flexibility regarding the blinding will be needed to accommodate inevitable feasibility constraints [12].

### Equivocal nodal lesions

Nodal lesions need to be discussed independently. In version 1.1 of the guidelines drafted by the RECIST workgroup, it was decided to set a size limit of 10 mm in the short axis for “pathological” lymph-nodes [13]. The risk of selecting a non-malignant lymph-node within the initial pool of lesions differs between targets and non-targets.

- Equivocal lymph nodes as Targets

The measurability threshold of these pathological nodes is specifically set higher (i.e. 15 mm vs 10 mm for non-nodal) which greatly limits the overlap range with a physiological lymph node even if the risk is not null.

Moreover, for lymph nodes, it can be noted that if they prove to be reactive (i.e., without tumor cells), there is a greater chance of their being inflamed in reaction to the cancer. In this case, their evolution should follow the course of the disease without distorting the response.

- Equivocal lymph nodes as Non-Targets

Unfortunately, what is valid for measurable nodes is not valid for nodes in the non-measurable category (i.e., 10–15 mm). Indeed, the 10 mm size criterion used to characterize a "pathological" lymph node is not highly specific and this choice is very debatable. It is not uncommon to find chronic centimetric lymph nodes in smokers at mediastinal level (Fig. 1d). Conversely, in the neck, a necrotic subcentimetric lymph node is unequivocally specific in ENT carcinomas.

The 10–15 mm lymph nodes are then at high risk of being classified as non-malignant NTL (in the absence of other criteria such as shape, extra-nodal extension signs and density) [14].

### Fewer targets, higher risk

In most studies, the measurability of the tumor features in the inclusion criteria to enable a quantified assessment. However, it is not uncommon for patients to have a single measurable lesion at baseline examination [2]. In this case, as previously demonstrated, the fewer the lesions available, the higher the impact of including an ambiguous lesion.

Technically speaking, in extreme cases, if a single lesion is chosen and is found to be a NMTL, a single reading paradigm will technically simulate a stable disease (SD) response. In this case, the double-reading paradigm usually challenges this SD status versus a Non-Disease status (if the single lesion is not selected) or a Non-Measurable disease status (if the single lesion is classified as NTL by the second radiologist).

In addition, it is important, for example, to highlight that some adjuvant trials might include patients with only non-measurable disease or no disease at all (after surgery or radiation). In this case, the simulation described above applies only to NTL and not to TL.

### Adaptive strategy according to the assessment endpoint

Awareness of the impact resulting from inclusion of a non-malignant lesion will help the radiologist to make selections in a more informed manner, especially in clinical trial assessments.

Two types of surrogate endpoints are derived from the oncologic assessment, i.e., tumor shrinkage and tumor progression. Usually, they are defined according to the primary outcome of the study and differ between phase 2 (response-related endpoints) and phase 3 (progression-related endpoints) trials.

Once the primary outcome has been defined, the radiologist’s can choose at baseline between a conservative versus a non-conservative approach regarding lesion management (Table 3).

### A centralized review also mitigates the risk

The preventive actions listed above are classic procedures in study management. Despite this, ambiguous lesions persist at baseline selection and the reading process, image presentation and reading paradigm continue to play a key role in mitigating the risk posed by equivocal lesions.

Regarding the reading process, during independent studies, radiologists should not be allowed to retrospectively revoke a target without clear justification. The FDA recommendations encourage the use of an image-lock approach for the reading process whereby readers interpret the assigned image and lock their reads (e.g., lesion measurements, response category, lesion severity) [7]. One possible modification to previous image interpretations would be to allow radiologists to subsequently eliminate a lesion that turns out to be a scar.

Several more efficient modes of image presentation can be used for RECIST evaluation. Notably, simultaneous presentation of all the time-points would prevent equivocal situations resulting from analysis of the baseline alone. Unfortunately, this is not always possible with ongoing studies.

Regarding the reading paradigm, a trial methodology named Blinded Independent Central Review (BICR) is commonly adopted to limit bias. In this setup, in the event of discrepant opinions between the two readers, the adjudicator can play a major role and mitigate the risk of reliance on a single independent non-conservative reader. Thanks to the adjudicator’s overall retrospective assessment of the entire study, non-malignant targets selected at baseline can easily be detected.

## Conclusion

Although no studies have investigated the frequency of inclusion of equivocal lesions at baseline when applying RECIST, from radiologists’ experience, such inclusions are not uncommon. Despite preventive strategies designed to limit such situations, in some lesions, the distinction between malignant and non-malignant is difficult to make.

Making an inappropriate decision on this type of baseline equivocal lesion is mitigated by adjudication via a double-reading centralized review. However, centralized reviews intrinsically give rise to equivocal situations, particularly if the transfer of clinical information is not carefully managed.

In practical terms, the general conclusion of our simulations tends to show that selecting an equivocal lesion during the baseline selection for the RECIST evaluation most often impacts the Response-Related Endpoint (best overall response, overall response rate, duration of response, response rate).

Regarding progression-related endpoints, inclusion of a non-malignant lesion in the targets is risky, especially when there are few targets of the same size. One should bear in mind that the impact is maximized by the percentage of NMTL/sum but also by the first response before a PD. However, selecting a single NMTL would not impact the date of progression otherwise triggered by the occurrence of a new lesion or the progression of NTL.

For these reasons, in the context of a phase II trial, we would recommend strategizing a strictly conservative approach when analyzing the baseline (not recording any equivocal lesion). In contrast, in a phase III trial setting, recording an equivocal lesion as NTL is a winning strategy which will maximize the chances of detecting disease progression and avoid bias.

## Data Availability

Data and material used for the simulations are available on request.

## Abbreviations

BICR: Blinded independent central review
CR: Complete response
DOP: Date of progression
ENT: Ears, nose, throat
FDA: Food and drug administration
NMTL: Non-malignant target lesions
NTL: Non-target lesion
PD: Progressive disease
PR: Partial response
RECIST: Response evaluation criteria in solid tumors
SD: Stable disease
SoD: Sum of diameter
TL: Target lesion
